# Development of antiviral and bacteriostatic chitosan‐based food packaging material with grape seed extract for murine norovirus, *Escherichia coli* and *Listeria innocua* control

**DOI:** 10.1002/fsn3.1910

**Published:** 2020-09-30

**Authors:** Collins Amankwaah, Jianrong Li, Jaesung Lee, Melvin A. Pascall

**Affiliations:** ^1^ Department of Food Science and Technology The Ohio State University Columbus OH USA; ^2^ Department of Veterinary Biosciences The Ohio State University Columbus OH USA

**Keywords:** antimicrobials, bacteria, Chitosan, grape seed extract, viruses

## Abstract

Edible coatings and films based on chitosan, and containing grape seed extract (GSE), were developed and their activities tested against murine norovirus (MNV‐1), *Listeria innocua* and *Escherichia coli* K12. Grape seed extract concentrations of 1%, 1.5%, and 2.5% dissolved in deionized water resulted in MNV‐1 plaque reductions (*p* < .05) of 1.75, 2.60, and 3.58 log PFU/ml, respectively after 3 hr. Two percent (w/w) chitosan solutions incorporated with 2.5% and 5% GSE also significantly (*p* < .05) reduced MNV‐1 titers by 2.68 and 4.00 log PFU/ml, respectively after 3 hr. Additionally, incorporation of the GSE into the chitosan films also showed antimicrobial efficacy against MNV‐1, *L*. *innocua,* and *E*. *coli* K12. Chitosan films containing 5%, 10%, and 15% GSE caused MNV‐1 reductions of 0.92, 1.89, and 2.27 log PFU/ml, respectively, after 4 hr of incubation. Also, after 24 hr, the 5% and 10% GSE films reduced MNV‐1 titers by 1.90 and 3.26 log PFU/ml, respectively, while the 15% GSE film reduced MNV‐1 to undetectable levels. For *E*. *coli* K12, there were reductions of 2.28, 5.18, and 7.14 log CFU/ml after 24 hr exposure by the 5%, 10%, and 15% GSE films, respectively. Also, *L*. *innocua* counts were reduced by 3.06, 6.15, and 6.91 log CFU/ml by the 5%, 10%, and 15% GSE films, respectively. This study demonstrated that GSE in edible films and coatings is effective against the organisms tested, and this shows that they are effective against foodborne microbes of public health concerns.

## Graphical abstract

This study investigated the use of an edible chitosan film impregnated with grape seed extract for the control of foodborne viruses and bacteria. The study showed that 15% grape seed extract significantly limited the growth of these organisms.

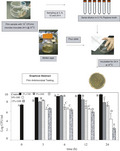

## 1 INTRODUCTION

Food contamination with pathogenic microorganisms is a serious threat to food safety and public health. Increased worldwide consumption of fresh, minimally processed, and ready‐to‐eat foods and lifestyle changes are among factors driving high levels of foodborne illnesses. Ready‐to‐eat foods can easily become cross contaminated or re‐contaminated with foodborne pathogens by food handlers and equipment surfaces during processing or packaging. Although various intervention technologies have been used to limit incidences of this menace, the problem continues. Antimicrobial packaging is one such technique that has been used to control foodborne illnesses. However, the focus of this effort revolved mainly on antibacterial and antifungal packaging. This paper reports on efforts to use food packaging as an antimicrobial tool against a foodborne virus.

Human norovirus is a common foodborne virus that belongs to the Caliciviridae family. It causes gastroenteritis, symptoms of which include vomiting, diarrhea, nauseas, abdominal cramps and fever. The virus is highly contagious and can be transmitted through the fecal‐oral route. Also, enteric foodborne bacteria such as *Escherichia coli* O157:H7 and *Listeria monocytogenes* can also cause illnesses. *Escherichia coli* O157:H7 is gram‐negative and has been associated with foodborne diseases such as bloody diarrhea, hemolytic uremic syndrome, and hemorrhagic colitis (Griffin & Tauxe, 1991). Outbreaks of E. *coli* O157:H7 are normally associated with bovine products; however, other food products such as leafy greens have been linked to this bacterium (Feng, 1995). *Listeria monocytogenes* is a ubiquitous microorganism and can be found in environments such as soil, vegetation, and in animals. It is a gram‐positive facultative anaerobe and is an intracellular bacterium that causes listeriosis. This is a potentially lethal disease in immune‐compromised individuals such as the elderly, pregnant women, and infants (Schuppler & Loessner, 2010). *L. monocytogenes* is also able to grow at refrigerated temperature and this makes food stored in this environment susceptible to this organism (Santos et al., 2019).

Interests in edible films are on the increase because, in addition to reducing environmental waste, they also serve as carriers of functional additives such as antimicrobial agents. These naturally derived extracts are safe to use in food and are excellent sources of natural phenolic compounds that have antimicrobial activities. Grape seed extract (GSE), (*Vitis vinifera*) as an example, can have antifungal, antiviral, and antibacterial activities. Recently, it was reported that GSE can inactivate murine norovirus (MNV‐1), a surrogate for human norovirus (Li, Baert, Zhang, Xia, Zhong, Van Coillie et al., 2012; Su & D’Souza, 2011). The antibacterial activities of GSE have also been demonstrated against *Listeria monocytogenes*, *E. coli* O157:H7, *Salmonella typhimurium,* and *Staphylococcus aureus* (Baydar et al., 2006; Kao et al., 2010; Rhodes et al., 2006). Corrales et al. (2009) incorporated GSE into a pea starch film and showed that 1% GSE reduced the bacterial growth on pork loins by 1.3 log CFU/ml after 4 days at 4°C.

Edible films and coatings with added antimicrobial agents have been demonstrated to have efficacies against several foodborne bacteria (Martins, Cerqueira, & Vicente, 2012; Siripatrawan & Harte, 2010; Siripatrawan & Noipha, 2012). Since the use of edible films and coatings to control foodborne viruses has not been explored, this study will investigate the use of chitosan film forming solutions (FFS) and films enriched with GSE for their virucidal activities on MNV‐1, *L. innocua,* and *E*. *coli* K12.

## 2 MATERIALS AND METHODS

### 2.1 Materials

Murine norovirus strain (MNV‐1) used as a human norovirus surrogate was obtained from Herbert W. Virgin IV, Washington University School of Medicine. The murine macrophage cell line RAW 264.7 (ATCC) was used to grow the MNV‐1. High‐glucose Dulbecco's Modified Eagle Medium (DMEM) and Fetal Bovine Serum (FBS) were purchased from Invitrogen and used for growing the cells. Six‐well plates were obtained from Corning Life Sciences and used to culture cells for plaque assay. *Escherichia coli* K12 (ATCC 29181) and *Listeria innocua* (ATCC 33090) were purchased from the American Type Culture Collection. Tryptic soy broth (TSB) and tryptic soy agar purchased from Difco were used to grow the bacteria. Medium molecular weight chitosan powder (94% purity, 75% deacetylation) was obtained from Huantai Goldenlake Carapace Products Co., Ltd and used to form the films. Glycerol was obtained from Fisher Scientific and used as a plasticizer. Glacial acetic acid was purchased from J.T Baker and used as a solvent. Leucoselect^®^ contained ≥ 95.0% ≤105.0% proanthocyanidins, as determined by Gel permeation chromatography, and ≥ 3.0% ≤19.0% catechin and epicatechin, as determined by high‐pressure liquid chromatography.

### 2.2 Film formation

**Figure 1:**
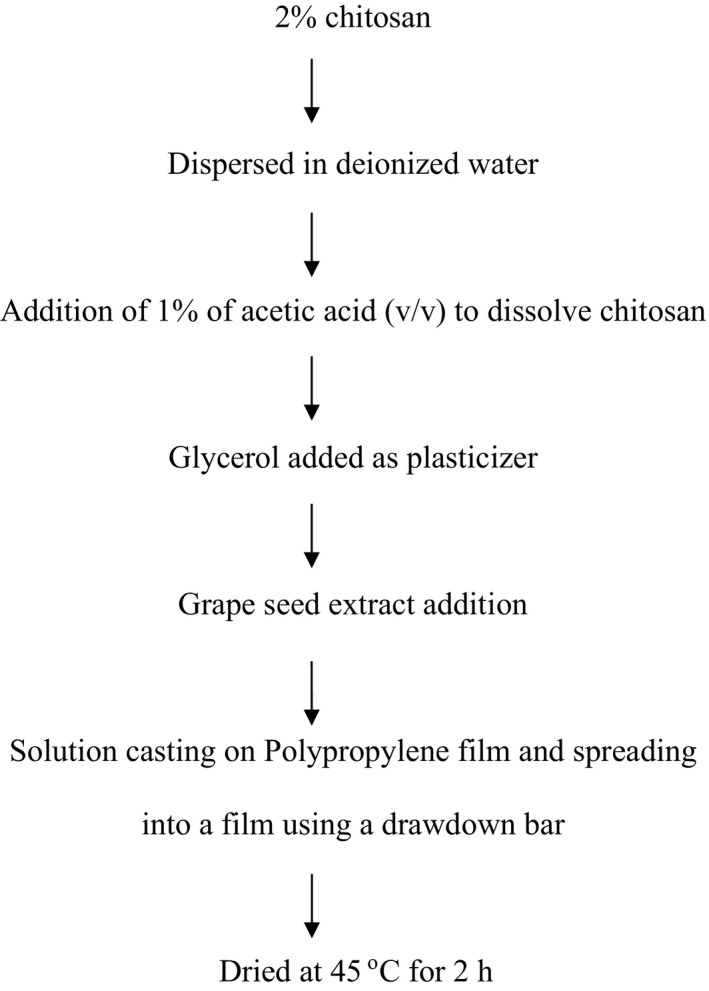
Flow chart of chitosan/GSE blend and film formation

To make the films, chitosan (2% w/w) was dissolved in 1% acetic acid solution with glycerol as a plasticizer (Figure 1). Grape seed powder at different concentrations (0%–15%) was added to the chitosan solution (Table 1). The solutions were then stirred using a magnetic stirrer for 30 min to dissolve the extract. The FFS was degassed in a water bath sonicator. A 20 g aliquot of the solution was poured onto a polypropylene sheet attached to a glass plate and a drawdown bar used to spread the solution into a thin film. After drying in an oven at 45°C for 2 hr, the films were peeled and stored for further testing. The film thickness from each formula was measured with a micrometer (Mitutoyo Manufacturing Co. Ltd.). The thicknesses of the films were averages from five random readings and are shown in Table 1.

**Table 1:** Formulations and thicknesses of chitosan‐based grape seed extract (GSE) antimicrobial films

Antimicrobial films	Chitosan (g)	Glycerol (g)	GSE (g)	Deionized water (ml)	Film thickness (mm)
Chitosan only	2	0.6	‐	100	0.024 ± 0.002
5% GSE film	2	3.5	5	100	0.063 ± 0.009
10% GSE film	2	6.5	10	100	0.073 ± 0.005
15% GSE film	2	8.5	15	100	0.113 ± 0.005

### 2.3 Virus stock preparation

The MNV‐1 was grown in a monolayer of RAW 264.7 cell line cultured in DMEM that was supplemented with 10% FBS at 37°C under a 5% CO_2_ atmosphere. Confluent RAW 264.7 cells were infected with MNV‐1 at a multiplicity of infection (MOI) of 1. After 1 hr of incubation at 37°C, 20 ml of serum‐free DMEM was added and incubated at 37°C under 5% CO_2_. The virus was harvested after 2 days of incubation by three freeze–thawing cycles and centrifuging at 3,000 g for 20 min at 4°C. The supernatant was collected and used as a stock.

### 2.4 Quantification of virus titer by plaque assay

The MNV plaque assay was performed in RAW 264.7 cells seeded into six‐well plates and incubated for 24 hr at 37°C under a 5% CO_2_ atmosphere (Wobus, Thackray, & Virgin, 2006). The cells monolayers were then infected with 400 µl of appropriate serial dilutions of the virus suspension and the plates incubated for 1 hr at 37°C and shaken slightly every 15 min to allow for the virus attachment. After removal of the inocula, the cells were overlaid with 2 ml of Eagle Minimum Essential Medium (MEM) containing 1% agarose, 2% FBS, 1% sodium bicarbonate, 0.1 mg of kanamycin/ml, 0.05 mg of gentamicin/ml, 15 mM HEPES (pH 7.7), and 2 mM L‐glutamine (Invitrogen). After 48 hr of incubation at 37°C and 5% CO_2_, the plates were fixed and stained with 10% formaldehyde and crystal violet. The plaques were then counted.

### 2.5 Virucidal/antiviral testing of GSE in aqueous and FFS

The GSE was dissolved in deionized water and sterilized by passing through a 0.2 µm filter. Concentrations of up to 2.5% GSE dissolved in deionized water (aqueous solutions) were tested by adding equal volumes of the virus suspension (10^7^ PFU/ml). Volumes of 0.5 ml of both the virus suspensions and aqueous extract solutions were mixed to give final extract concentrations of 1.0%, 1.5%, and 2.5%. The mixtures were incubated at 23 ± 1°C on a rotary shaker for 3 hr. For the control, an equal volume of DMEM was mixed with the virus suspension. After 3 hr, 0.5 ml of the treated samples and the control were withdrawn and each diluted with 0.5 ml DMEM. After 10‐fold serial dilutions, the appropriate solutions were plaque assayed as previously described.

A similar procedure was followed for testing reductions in the virus infectivity by the FFS. To perform this test, chitosan solutions containing 5% and 10% GSE were mixed with equal volume of virus suspensions to yield final concentrations of 2.5 and 5.0% GSE, respectively. The controls were DMEM and 2% chitosan solution mixed with equal volumes of the virus suspensions.

### 2.6 Virucidal/antiviral testing of chitosan/GSE films

In testing the virucidal activity of the films, the method of Haldar, An, Álvarez de Cienfuegos, Chen, and Klibanov (2006) was used with slight modification. Film samples containing the different levels of GSE (5%, 10%, and 15%) and measuring 2.5 cm × 2.5 cm were individually placed in the bottom of one of the wells of a 6‐well plate and 2 ml of the virus suspension (~10^7 ^PFU/ml) added to each film. They were then incubated at 23 ± 1°C on an incubator shaker for 24 hr. Virus suspensions with no added film and one with 2% chitosan film but with no added extract were used as controls. After 4 and 24 hr, 0.5 ml of the treated samples and controls were withdrawn and diluted with 0.5 ml of DMEM. Ten‐fold serial dilutions were then made, and appropriate dilutions plaque assayed as previously described. The experiments were done in duplicates.

### 2.7 Antibacterial activity testing of films

For the bacterial testing, only the antimicrobial films were used. The bacterial species were individually cultured by transferring a loopful of frozen (−80°C in 30% glycerol) *E. coli* K12 and *L. innocua* into 20 ml of sterile TSB with an inoculation loop. The organisms were incubated for 24 hr at 37°C to reactivate them. Afterward, a loopful of each revived bacterium was transferred to a TSA slants and incubated for 24 hr at 37°C. The slants were then refrigerated at 4°C and used as a stock culture. Before each experiment, a loopful of the respective bacterium was taken from the slant and cultured in a 20 ml sterile TSB for 24 hr at 37°C. Bacterial suspensions containing approximately 10^7^ CFU/ml were prepared from the overnight culture and used for the antibacterial testing of the film.

A similar method used for the virucidal/antiviral testing was also used to quantify the bacterial survival. Film samples measuring 2.5 cm × 2.5 cm were placed in a dish to which 2 ml of each (10^7^ CFU/ml) bacteria suspensions added. Cultures without a film and another with the 2% chitosan film but no added extracts were used as controls. The dishes were incubated at 37°C and shaken at 60 rpm in an incubator shaker for 24 hr. Samples were taken at 0, 3, 6, 12, and 24 hr intervals and diluted with 0.1% peptone solutions. Appropriate dilutions were pour‐plated using TSA in duplicates. The plates were incubated for 36 hr at 37°C and the number of colonies counted using a Darkfield plate counter (American Optical).

### 2.8 Statistical analysis

All data were analyzed using the analysis of variance (ANOVA), and Tukey's multiple comparisons test was used to determine the antimicrobial efficacies of different concentrations of the GSE in the solutions and in the dried chitosan films. The level of significance was set at *p* < .05. The statistical package used in the study was IBM SPSS for Windows. The statistical analysis was used to compare and determine the significant effect of the addition of GSE on: (1) the antiviral efficacies of different concentrations of GSE dissolved in deionized water and chitosan FFSs on MNV‐1; (2) the antiviral and antibacterial efficacies of chitosan films containing 3 levels (5%, 10% and 15%) of GSE on MNV‐1, and *E*. *coli* K12 and *L*. *innocua*, respectively. All data presented are the means of four replications.

## 3 RESULTS

### 3.1 Antiviral effects of GSE in water and the chitosan FFS

**Figure 2:**
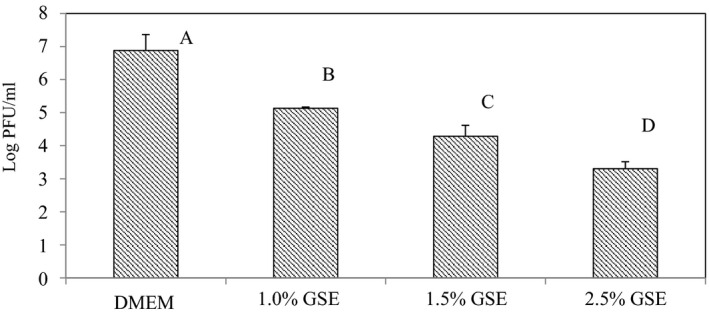
Infectivity of MNV‐1 after treatment with different levels of aqueous solution of grape seed extracts (GSE) and the Dulbecco's Modified Eagle Medium (DMEM). Reduction in MNV‐1 infectivity was detected by plaque assay after 3 hr of incubation at 23 ± 1°C. Error bars indicate standard deviation

**Figure 3:**
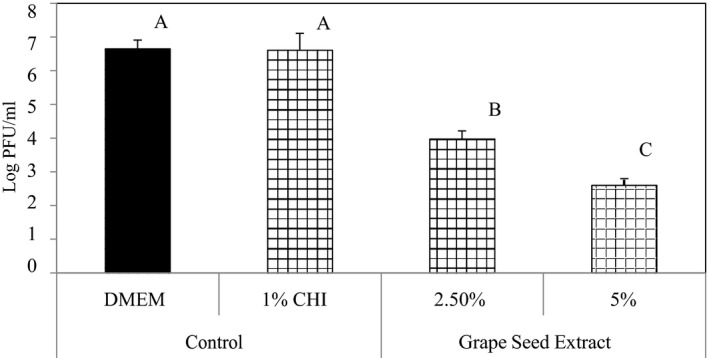
Infectivity of MNV‐1 after treatment with chitosan (CHI) film forming solutions containing grape seed extracts and Dulbecco's Modified Eagle Medium (DMEM). Reduction in MNV‐1 infectivity was detected by plaque assay after 3 hr of incubation at 23 ± 1°C. Error bars indicate standard deviation

Figure 2 shows the results of the virucidal effects of GSE against MNV‐1 when dissolved in deionized water (aqueous extract solutions). The results show that from the initial count of ~10^7^ PFU/ml, log reductions of 1.75, 2.60, and 3.58 log PFU/ml were obtained by the 1.0%, 1.5%, and 2.5% GSE aqueous solutions, respectively after 3 hr. The antiviral/virucidal efficacy of the extracts was further assessed when they were incorporated into the chitosan FFS. The results show that the 2.5% and 5.0% GSE samples presented in Figure 3 produced MNV‐1 titer reductions of 2.68 and 4.00 log PFU/ml, respectively. Both Figures 2 and 3 show that the log reductions were statistically significant (*p* < .05).

### 3.2 Antiviral/virucidal effect of the GSE films

**Figure 4:**
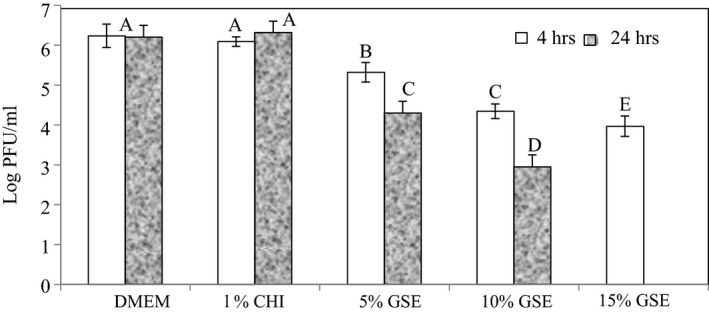
Infectivity of MNV‐1 after treatment with chitosan (CHI) films with different levels of grape seed extracts. Reduction in MNV‐1 infectivity was detected by plaque assay after 4 and 24 hr of incubation at 23 ± 1°C. Error bars indicate standard deviation

In Figure 4, the results of the chitosan films blended with the different levels of GSE are presented. The results show that they significantly (*p* < .05) reduced MNV‐1 titers. The films containing 5%, 10%, and 15% GSE produced reductions of 0.92, 1.89, and 2.27 log PFU/ml, respectively, after 4 hr incubation. After 24 hr of incubation, titer reductions of 1.90 and 3.26 log PFU/ml were obtained for the 5% and 10% GSE films, respectively. The 15% GSE film reduced the MNV‐1 titers to undetectable levels after 24 hr.

### 3.3 Antibacterial activity of the GSE films

**Figure 5:**
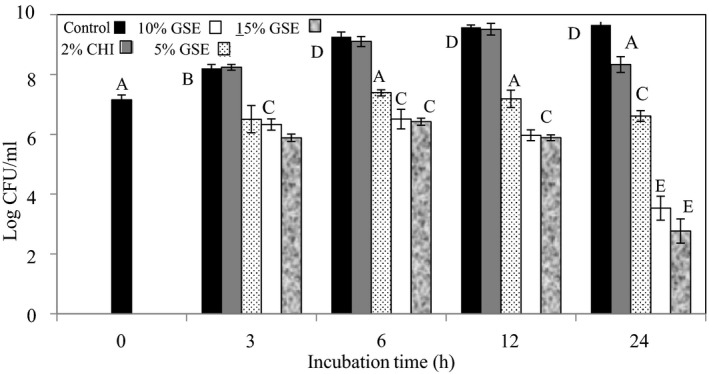
The antibacterial activity of chitosan films incorporated with grape seed extract. The effect of grape seed extracts incorporated into chitosan films on the survival of *Listeria innocua* was determined in tryptic soy broth at 37°C. Error bars indicate standard deviation

**Figure 6:**
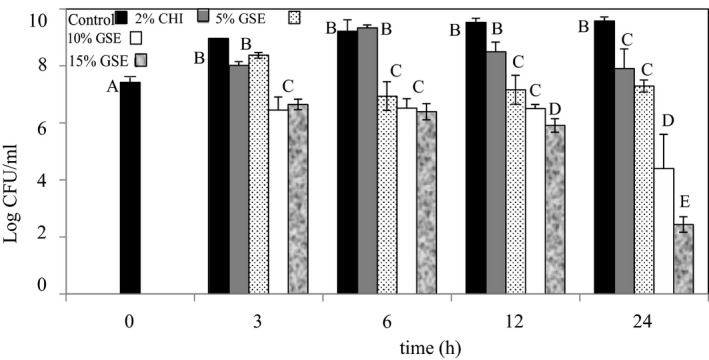
The antibacterial activity of chitosan films incorporated with grape seed extract. The effect of grape seed extracts (GSE) incorporated into chitosan (CHI) films on the survival of *Escherichia coli* K12 was determined in tryptic soy broth at 37°C. Error bars indicate standard deviation

The antibacterial efficacies of the GSE films were also investigated against *L. innocua* and *E*. *coli* K12. In conducting the experiment, two different controls were tested. These were the one having no added GSE extract and the other having 2% chitosan and no added GSE extract. Figures 5 and 6 summarize the results obtained. The bacterial viabilities were assessed after 0, 3, 6, 12, and 24 hr of incubation. After 3 hr of incubation, the 5, 10, and 15% GSE films reduced *L*. *innocua* counts significantly (*p* < .05) by 1.70, 1.88, and 2.32 log CFU/ml, respectively, when compared with the controls. Although the GSE films reduced the *L*. *innocua* load after 6 hr, when compared with the results at 3 hr incubation, there was no significant difference (*p* > .05) in the counts produced by the 10% and 15% GSE films, which recorded reductions of 2.75 and 2.84 log CFU/ml, respectively. The 5% GSE film produced 1.88 log CFU/ml reductions of *L*. *innocua* after 6 hr. After 12 hr incubation, the 5% GSE films produced *L*. *innocua* reductions of 2.39, 3.60, and 3.69 log CFU/ml. These were also not significant (*p* > .05) from the results obtained at 6 hr incubation. While there were significant differences (*p* < .05) between the 5 and the 10% GSE films, the results between the 10 and 15% were not significantly different (*p* > .05). For the 24 hr incubation, reductions of 3.06, 6.15, and 6.91 log CFU/ml by the 5, 10, and 15% GSE films, respectively, were obtained. These results were significantly (*p* < .05) higher than those obtained at 12 hr incubation.

The results obtained for *E*. *coli* K12 followed a pattern similar to that for the *L*. *innocua*. At 3 hr incubation, reductions of approximately 2 log cycles were recorded for the 10% and 15% GSE films. After 6 hr of incubation, reductions of 2.28, 2.70, and 2.82 log CFU/ml were recorded by the 5%, 10%, and 15% GSE films, respectively. There were no significant differences (*p* > .05) between the results obtained for the GSE films at 6 hr of incubation for the 10% and 15% GSE films although the 5% films between the 3 and 6 hr incubation were significantly different (*p* < .05). However, the GSE films significantly (*p* < .05) reduced *E*. *coli* K12 levels when compared with the results for the control. Figure 6 shows that the 5%, 10%, and 15% GSE films significantly (*p* < .05) reduced the *E*. *coli* K12 population by 2.36, 3.02, and 3.62 log CFU/ml, respectively, after 12 hr when compared with the controls. However, only the 15% GSE film was significantly different between the 6 and 12 hr incubation period. This significant reduction was also obtained after 24 hr of incubation when compared with the 12 hr result. Figure 6 shows that at 24 hr, the films containing 5% GSE reduced *E*. *coli* K12 counts by 2.28, the 10% GSE film by 5.18, and the 15% GSE film by 7.14 log CFU/ml.

## 4 DISCUSSION

Nonenveloped viruses tend to be resistant against commonly used preservatives in the food industry (Li, Baert, Zhang, Xia, Zhong, Jiang et al., 2012). In this study, we evaluated the antiviral/virucidal activity of GSE. Since GSE is known to enhance the inactivation of foodborne bacterial pathogens, our main interest was to see if it can also inactivate MNV‐1. Therefore, we incorporated GSE into chitosan FFS and films and tested them for their antiviral/virucidal activities. Aqueous GSE solutions were also tested. From the results obtained, the aqueous GSE solutions were capable of inactivating the MNV‐1. However, when incorporated into the chitosan FFS, a higher concentration of the GSE (2.5%) was needed to produce a similar MNV‐1 reduction when compared with the GSE alone (1.5%). This seems to indicate that interactions between the chitosan and the GSE in the aqueous solution made the GSE less efficient as an antimicrobial agent against the MNV‐1. When the dried chitosan/GSE films were produced, the results showed that the chitosan/GSE significantly enhanced the antimicrobial effectiveness of the film against MNV‐1 when compared with the chitosan alone.

Chitosan is widely reported to have antimicrobial properties against selected bacteria and fungi, but little is reported on its antimicrobial activity against viruses. Grape seed extracts are rich in oligomeric proanthocyandins (OPCs) which are the dimeric, trimeric, and tetrameric forms of the catechins. Su and D'Souza (2011) studied the effect GSE against viral surrogates, including MNV‐1, FCV, Feline Calicivirus, and bacteriophage MS2 in solution and reported reductions of viral infectivity. Additionally, Iwasawa et al. (2009) demonstrated the effect of proanthocyanidins on feline calicivirus and coxsackie A7. Furthermore, Li, Baert, Zhang, Xia, Zhong, Jiang et al. (2012) recently confirmed the virucidal activity of GSE. These authors investigated the anti‐norovirus activity of GSE on MNV‐1, human norovirus virus‐like particles (VLP), human norovirus P particles, and human norovirus GII.4. Results reported by Li, Baert, Zhang, Xia, Zhong, Jiang et al. (2012) included reductions in the binding levels of the human norovirus GII.4 and the P particles. They also reported MNV‐1 titers reductions after incubation with GSE. When transmission electron microscopy (TEM) was used to assess the morphology of the VLP, the structure of the untreated VLP sample was spherical in shape. However, the GSE‐treated VLP samples had deformations and inflations which signified that the GSE caused damages to the capsid proteins (Li, Baert, Zhang, Xia, Zhong, Jiang et al., 2012). Su and D'Souza (2011) also reported that GSE is capable of affecting viral adsorption and replication using pre‐ and postinfection viral studies, respectively. Other polyphenols and polyphenol‐rich products such as pomegranates, cranberry juice, and gallic acid have also reduced titers of norovirus surrogates (Howell & D’Souza, 2013; Joshi et al., 2016; Su & D'Souza, 2011). The mechanism(s) responsible for the antiviral/antiviral activity of GSE therefore included disruption of capsid protein, interfering with viral attachment/adsorption and/or interfering with viral replications. What is different with these studies and our study is the fact that we incorporated the GSE into chitosan and used it to make edible films. Our interest was to determine whether the GSE would still shown antimicrobial, and antiviral properties in particular, when blended with the chitosan and made into films. Since chitosan and GSE are derived from foods normally ingested by the consuming public, our concern was to develop an edible polymeric film with antimicrobial, and antiviral properties in particular.

The quest for antimicrobial agents with broad spectral activities has resulted in an increased interest in the use of naturally derived plant extracts. Additionally, consumers are increasingly demanding foods that are free from synthetic chemicals. In this study, the antibacterial activities of the GSE films were also tested against *L*. *innocua* and *E*. *coli* K12. Here too, GSE addition enhanced the efficacy of the edible films. Corrales et al. (2009) incorporated 1% GSE into a pea starch film. The results showed a reduction in the growth of *Brochothrix thermosphacta* on pork loins by 1.3 log CFU/ml after 4 days at 4°C. Additionally, Moradi et al. (2011) reported that 1% GSE incorporated into chitosan films reduced aerobic mesophilic and lactic acid bacteria populations on sausage by 1.1 and 0.7 log CFU/ml respectively, after 21 days of storage. In yet another example, Gadang et al. (2008) incorporated 3% GSE into protein coatings and reported a reduction of *Salmonella typhimurium* counts by 2 log CFU/ml. In some instances, GSE is used in combination with other preservatives such as nisin, oregano oil, EDTA, and malic acid (Gadang et al., 2008; Moradi et al., 2011; Theivendran et al., 2006). For instance, soy protein edible films containing 1.0% GSE combined with 1.0% nisin resulted in 2.8 log CFU/ml reductions against *L. monocytogenes* in turkey frankfurter (Theivendran et al., 2006). In this present study, high levels of GSE (15%) were incorporated into the chitosan films and this enhanced the film's efficacy against MNV‐1 (virus), *L*. *innocua,* and *E*. *coli* K12 (bacteria).

In explaining the mechanism of action for GSE, Perumalla and Hettiarachchy (2011) stated that the partial hydrophobic nature of polyphenolic compounds enhances their accumulation and attachment to the bacterial cytoplasmic membrane. Therefore, polyphenolic compounds are expected to initiate antibacterial activity through interactions with the outer cell membrane of the bacterium. The antibacterial efficacies of these polyphenols correlate with the presence of reactive groups such as the hydroxyls, galloyl moieties, and conjugated double bonds (Ikigai et al., 1993; Ultee, Bennik, & Moezelaar, 2002). These reactive groups are capable of enhancing the efficacies of the polyphenols. Consequently, the interaction of these active groups with the cell membrane would lead to cell death through the physical disruption of the membrane, dissipation of the proton motive force (PMF), and/or inhibition of membrane‐associated enzymatic activities (Juven et al., 1994; Shimamura et al., 2007). This increases the permeability of the membrane and results in leakage of the cellular constituents. Once the membrane is damage, the polyphenols can also enter into the interior of the cell and subsequently impair enzymatic activities and destroy genetic materials (Ikigai et al., 1993). Also, chitosan by itself is known to inhibit bacterial growth by increasing cell permeability, interacting with DNA and messenger RNA and acting as a chelating agent that binds metals and nutrients (Martınez‐Camacho et al., 2010; Rabea et al., 2003; Tripathi et al., 2010).

## 5 CONCLUSIONS

It can be concluded from this study that GSE was successfully incorporated into chitosan‐based films. Also, that GSE had antiviral/virucidal and antibacterial efficacies when incorporated into the chitosan films. When in the FFS, GSE was also effective as an antimicrobial agent. This showed that compounds in the film solution did not significantly interfere with the antimicrobial properties of the GSE.

The conclusion could be made that a 15% GSE/chitosan film would be needed to significantly reduce MNV‐1, *L. innocua,* and *E. coli* K12 after 24 hr incubation.

## CONFLICT OF INTEREST

The authors declare that they do not have any conflict of interest.

## AUTHOR CONTRIBUTIONS

Saeid Hazrati, Seyyed Jaber Hosseini, and Silvana Nicola: were involved in designed and conducted the research, data collection, analysis of results, and writing‐original draft manuscript. Saeed Mollaei, Hossein Rabbi Angourani, Seyyed Jaber Hosseini, and Mojde Sedaghat: were involved in development of the study design and data curation. Saeid Hazrati, Seyyed Jaber Hosseini, and Saeed Mollaei: were involved in development of the analysis of results and contributed to submitting the manuscript. Saeid Hazrati, Mojde Sedaghat: and Silvana Nicola reviewed and revised the manuscript.

## ETHICAL APPRORAL

This study did not involve any human or animal testing.

## INFORMED CONSENT

Written informed consent was obtained from all study participants prior to submitting the manuscript for publication.

## Data Availability

The data that support the findings of this study are available from the corresponding author upon reasonable request.
